# 2243. Patterns of Micafungin Prescribing in the Intensive Care Unit in an Observational Retrospective Cohort

**DOI:** 10.1093/ofid/ofad500.1865

**Published:** 2023-11-27

**Authors:** Francesca E Cozzi, Raul Rodriguez, Fischer Herald, Hayley A Hodgson, Sarah E Sansom, Shivanjali Shankaran, Christy A Varughese, Sarah Y Won

**Affiliations:** Rush University Medical Center, Chicago, Illinois; Rush University Medical Center, Chicago, Illinois; Rush University Medical Center, Chicago, Illinois; Rush University Medical Center, Chicago, Illinois; Rush University Medical Center, Chicago, Illinois; Rush University Medical Center, Chicago, Illinois; Rush University Medical Center, Chicago, Illinois; Rush University Medical Center, Chicago, Illinois

## Abstract

**Background:**

Micafungin is restricted by the Antimicrobial Stewardship group at Rush University Medical Center (RUMC), but use in intensive care units (ICUs) is higher than predicted according to benchmarking data. Micafungin is frequently requested empirically for sepsis despite the lack of evidence to support a mortality benefit. The objective of this review was to characterize micafungin requests in the ICUs at RUMC.

**Methods:**

Retrospective data was collected from June 2020 - August 2022 at RUMC, an academic tertiary care center in Chicago, IL with 104 adult ICU beds. Any patient with a new micafungin order during an ICU stay was reviewed for order appropriateness and clinical outcomes. Micafungin requests were classified as “empiric” or “documented candidiasis.” The primary outcome was incidence of fungemia on culture drawn 3 days before to 7 days after the micafungin order. Among empiric requests, incidence of invasive fungal infection (IFI), defined as fungi isolated in a culture from a sterile site, was also evaluated.

**Results:**

232 new micafungin orders were placed during the study period, of which 51 (22%) were prescribed for documented candidiasis and 181 (78%) were empiric. Of patients for whom empiric micafungin was ordered, 18/181 (10%) subsequently developed an IFI. Of these, only 6/181 (3%) developed fungemia. Other IFI identified included 8/181 (4%) abdominal infections and 4/181 (2%) with positive miscellaneous cultures. 90% (163/181) of patients with an empiric micafungin order did not develop an IFI.

**Conclusion:**

The overall incidence of critically ill patients who developed an IFI within 7 days of a micafungin request was very low. Data sharing, prescriber education, and additional stewardship interventions should be explored to improve the appropriateness of antifungal prescribing.

**Disclosures:**

**All Authors**: No reported disclosures

